# Spatial and Seasonal Patterns of Tick Infestations in Kassena-Nankana Livestock

**DOI:** 10.1155/2024/8889907

**Published:** 2024-01-09

**Authors:** Seth Offei Addo, Ronald Essah Bentil, Bernice Olivia Ama Baako, Jane Ansah-Owusu, Christopher Nii Laryea Tawiah-Mensah, Eric Behene, Victor Asoala, James C. Dunford, John Asiedu Larbi, Philip Kweku Baidoo, Michael David Wilson, Joseph W. Diclaro, Samuel K. Dadzie

**Affiliations:** ^1^Parasitology Department, Noguchi Memorial Institute for Medical Research, University of Ghana, Legon, Accra, Ghana; ^2^Department of Theoretical and Applied Biology, Kwame Nkrumah University of Science and Technology, Kumasi, Ghana; ^3^Navrongo Health Research Centre, Navrongo, Upper East Region, Ghana; ^4^Navy Entomology Center of Excellence, Jacksonville, Florida, USA; ^5^Uniformed Services University of Health Sciences, Bethesda, Maryland, USA; ^6^Navy Entomology Center of Excellence, Centers for Disease Control and Prevention Detachment, Atlanta, Georgia, USA

## Abstract

The ability of ticks to adapt to different ecological zones, coupled with the spread of infectious pathogens negatively affects livestock production and thus, there is a need for better control strategies. However, control measures within a geographical region can only be effective if there is available information on tick population dynamics and ecology. This study focused on ticks infesting livestock in the Kassena-Nankana Districts of the Upper East Region of Ghana. The ticks were morphologically identified, variables such as season, animal host, and predilection sites were recorded, and the data were analyzed using STATA version 13. Out of 448 livestock examined, tick infestation in cattle was (78.60%), followed by sheep (25%) and goats (5.88%). A total of 1,550 ticks including nymphs (303) and adults (1,247) were collected. Adult ticks were found to be significantly associated with season (*p* < 0.001), with a high burden in the wet season. The nymph burden and body parts of livestock hosts were significantly associated with more nymphs collected from male animals than females (*p* < 0.001). Three genera of ticks, *Amblyomma* (62.97%), *Hyalomma* (18.71%), and *Rhipicephalus* (18.32%) were morphologically identified with the most predominant tick species recorded as *Amblyomma variegatum* (62.97%). Matured *A. variegatum* was sampled primarily in the wet season with their predilection site as the udder/scrotum (*p* < 0.001). However, adult *Hyalomma truncatum* was observed to have a significant association with the anal region (*p* < 0.001). Findings from this study are essential for formulating tick control measures to prevent the spread of infectious pathogens.

## 1. Introduction

Ticks play a significant role in the transmission of infectious pathogens, acting as obligate blood-sucking vectors that cause not only skin damage but also facilitate the spread of diseases [1]. Although a majority of the tick-borne pathogens primarily affect livestock health, there are some zoonotic pathogens responsible for human disease [2, 3]. The Centers for Disease Control and Prevention (CDC) have reported a notable increase in tick-borne infections, a trend expected to continue [4]. This has raised much concern as control measures are ineffective and vaccines are limited [5]. In Africa, tick-borne diseases are particularly problematic, leading to livestock mortalities ranging from 10 to 80%, profoundly impacting farmers' livelihoods [6, 7]. Complex interactions between humans, environmental conditions, and biological factors make the management of these diseases challenging [8]. Also, these factors influence the tick burden on hosts [9], thus affecting the efficacy of tick control [10, 11]. With the level of tick infestation in animals serving as a focal point for controlling tick-borne diseases [7], it is essential to determine the animal hosts that are mostly infested [12] and their preferred resting sites on livestock.

Tick species' predilection for unique ecological conditions affects both their spread and the likelihood of tick-borne diseases that are connected with it [13]. Furthermore, ticks multiply and spread throughout an area by adjusting to animal and bird migratory patterns [14]. In Africa, livestock are mostly infested by hard ticks of the genera *Amblyomma*, *Hyalomma*, and *Rhipicephalus* [15]. These ticks have been identified in Ghana to harbour pathogens including Dugbe Virus [16] and Crimean-Congo Haemorrhagic Fever Virus (CCHFV) [17]. To control these tick species in Ghana, chemical control methods are often employed. However, there is very little information on the bionomics of ticks, seasonal variations in their distribution, and predilection sites on livestock.

The majority of people in Ghana's Upper East Region raise livestock for both food and profit. Some of these livestock are imported from Burkina Faso, increasing the risk of tick species invasion and the spread of infectious pathogens. The continuous trade of livestock which could be amplifying hosts for infectious zoonotic pathogens [18], coupled with potential tick vectors suggests the need to control prevailing tick populations. Again, increasing levels of tick infestation would negatively affect livestock production in the Kassena-Nankana Districts, leading to significant economic loss for small-scale farmers. In order to provide a baseline for the development and administration of effective tick control strategies, this study set out to evaluate the distribution of tick species and their site of preference on hosts.

## 2. Methods

### 2.1. Study Sites

Sites in the Upper East Regions' Kassena-Nankana Districts were chosen for this investigation. Livestock is emphasised as a significant economic activity in these sites. The districts' vegetation is Guinea Savannah, with two distinct seasons: the wet season, which runs from May to October, and the dry season, which runs from November to April. The majority of people living in the district engages in agriculture and raises livestock for both food and profit [19].

### 2.2. Ethical Approval

The University of Ghana Institutional Animal Care and Use Committee gave its approval to this study (UG-IACUC; UG-IACUC 001/19-20).

### 2.3. Sample Size and Tick Collection

Using Epi Info version 6, a required sample size of a minimum of 248 livestock was calculated.

The following criteria were used to calculate sample size: according to estimates from a local veterinarian, the livestock population was 5,000, with a predicted prevalence rate of 21.6% [20], a 5% error margin, and a 95% confidence level. The collection of ticks was done between February and December 2020, covering the wet and dry seasons. Three collections each were done in the wet and dry seasons. Ticks were manually removed from randomly chosen livestock, such as sheep, goats, and cattle, at each sampling location. Each animal was restrained and ticks were removed using blunt forceps from various sites of attachments on the host. Following their sorting according to the host's collecting site, the ticks were transferred to the Noguchi Memorial Institute for Medical Research and preserved in correctly labelled 2 ml Eppendorf tubes containing RNA. Using the given taxonomic keys, the ticks were identified morphologically in the laboratory [21].

### 2.4. Data Analysis

STATA version 13 was used to perform the statistical analysis. The relationship between tick burden and animal traits as well as season was examined using a univariate and multiple variable mixed effect negative binomial regression model. The negative binomial regression model was used to avoid overdispersion. The significance level was set at *p* < 0.05.

## 3. Results

### 3.1. Characteristics and Distribution of Ticks Collected

A total of 448 livestock were screened for tick infestation out of which 54.24% were cattle, 26.79% were sheep, and 18.97% were goats. The majority of the livestock were females (69.87%) and were below 4 years old (Table 1). In total, 1,550 ticks consisting of 303 nymphs and 1,247 adults were collected and identified. Nymphs were collected from only cattle with the majority infesting males (80.53%, *p* < 0.001) and cattle that were 3 years and younger (50.17%). From the risk analysis, male cattle (*p*=0.002) were at an increased risk of nymph infestation with the udder/scrotum (*p*=0.048) and chest (*p*=0.024) as the most preferred sites of attachment (Table 2).

Adult ticks were collected from mostly cattle (84.36%), followed by sheep (14.60%) and then goats (1.04%). It was observed that adult tick infestation was more common in female livestock (64.39%), livestock ≤3 years old (51.64%), and the ticks were often attached to the udder/scrotum (42.26%). It was observed that season is a risk factor for adult tick infestation with an increased burden in the wet season (*p* < 0.001) (Table 3).

### 3.2. Seasonal Distribution of Tick Species

The ticks identified were *Amblyomma variegatum* (62.98%), *Hyalomma rufipes* (10.9%), *Hyalomma truncatum* (7.81%), *Rhipicephalus evertsi evertsi* (12%), *Rhipicephalus* (*Boophilus*) sp. (5.35%), *Rhipicephalus geigyi* (0.9%), and *Rhipicephalus sanguineus* (s.l.) (0.06%).

Generally, more ticks were collected in the wet season (*n* = 837, 54%) compared to the dry season (*n* = 713, 46%). There was a significant difference in the distribution of adult *A. variegatum* in the wet season as compared to the dry season (*p* < 0.001). No significant differences were seen for the other identified tick species (Table 4). All the nymphs were collected in the dry season.

## 4. Predilection Sites of Tick Species

Out of the 1,550 ticks collected, the preferred sites of attachment on livestock were; udder/scrotum (*n* = 704, 45.42%), anal (*n* = 489, 31.55%), chest (*n* = 335, 21.61%), limbs (*n* = 18, 1.16%), and head (*n* = 4, 0.26%). Adult *A. variegatum* had a significant association with the udder/scrotum of sampled livestock (*p* < 0.001). However, adult *H. truncatum* was observed to have a significant association with the anal region (*p* < 0.001). There was no significant association between the other identified tick species and their preferred sites of attachment on the livestock host (Table 5).

## 5. Discussion

This study reports the occurrence of diverse tick species in the Kassena-Nankana Districts with *A. variegatum* as the predominant species. Cattle were found to be a suitable host for the majority of the tick species with the risk of adult tick infestation in the wet season. Furthermore, it was observed that generally, ticks preferred to attach and feed on the udder/scrotum of the livestock.

Tick infestation in the livestock from this study was recorded as 18.97% in goats, 26.79% in sheep, and 54.24% in cattle. This can be compared to a study in Pakistan that reported tick infestation to be 57.11% in cattle, 51.97% in sheep, and 46.94% in goats [22]. Again, it can be compared to a study in Senegal that reported tick infestation in cattle (92%), sheep (55%), and goats (13%) [23]. In this study, cattle were the most infested with diverse tick species amongst the sampled livestock. During the process of grazing, which often covers long distances, cattle within the study area are likely to be exposed to questing ticks [24] and subsequently infest other livestock when they return to their kraals. Findings from this study suggest that tick species have adapted to cattle as suitable hosts. The abundance and constant movement of cattle to new locations to graze allows ticks to complete their life cycle and disperse. There was no statistically significant correlation found between the adult tick burden and livestock sex, despite the fact that typically more females than males were tested. It was however observed that the tick nymph burden on male cattle was significantly high compared to the female cattle. This could be due to the extensive use of male cattle for farming activities and grazing over long distances which expose them to tick infestation [25, 26]. It was also observed that ticks did not have a preference for any specific age group of livestock although other studies have reported a low tick burden in younger livestock [27, 28].

Overall, *A. variegatum* was the predominant tick species found in this study infesting mostly cattle as has been shown in previous studies in Ghana [29–31]. It was further observed in this study that, whereas adult *A. variegatum* was found primarily in the wet/rainy season, nymphs were collected only in the dry season. This pattern has been observed in another study [32]. This finding suggests that matured *A. variegatum* often infest cattle during the rainy season [33], thus, cattle in the study areas are at an increased risk of tick infestation during this season. This would negatively affect livestock production in the Kassena-Nankana Districts; hence, there is a need to control *A. variegatum* populations. In Africa, *A. variegatum* has been reported in over 30 countries [21] and is the primary vector of *Ehrlichia ruminantium* [34, 35] and the spread of dermatophilosis [36] in livestock. In humans, these ticks transmit *Rickettsia africae* which causes African tick bite fever [37, 38]. *Amblyomma variegatum*, in addition to transmitting disease, can cause substantial blood loss from its hosts [39]. In severe infestations, the host may experience a decrease in appetite and weight, which puts them at risk for various diseases [40]. It is not uncommon for hundreds of ticks to be found on a single host [41].

*Rhipicephalus evertsi evertsi*, the most predominant *Rhipicephalus* species infesting sheep in this study are known to transmit *Babesia*, *Ehrlichia*, *Theileria*, *Anaplasma*, and *CCHFV* [42]. Previous studies have reported the distribution of this species in Ghana [29, 31], putting infested areas at risk of pathogen transmission. Furthermore, *Rhipicephalus* (*Boophilus*) sp. which prefers to feed on cattle [21] and *R. sanguineus* (s.l.), also known as brown dog tick [12] were identified although in low numbers. Nonetheless, these ticks spread pathogens including *Ehrlichia*, *Babesia*, and *Rickettsia* [43–45].

Only cattle were discovered to be infested by *Hyalomma rufipes* and *H. truncatum*, which were more common during the dry season than the wet one. This suggests an increased *Hyalomma* activity during periods of increased temperatures since these species actively chase suitable hosts [46]. The dry season in the study area is associated with less grass and vegetation, forcing cattle to spend more time and move over longer distances to graze. This creates the opportunity for tick species to locate hosts and attach for blood-feeding. Species of the genus *Hyalomma* maintain and transmit infectious pathogens like CCHFV in humans [47, 48] and *Theileria annulata* in cattle [49], especially within the tropical and subtropical regions [50]. In Ghana, CCHFV has been detected in *Hyalomma* with abattoir workers having been exposed to the virus indicating the public health importance of these tick species [17].

Generally, it was observed that tick species in the study area had a preference for mostly the udder/scrotum, followed by the anal and chest regions of hosts. The udder/scrotum is the safest and most conducive site for blood feeding as described by previous studies [51, 52]. The head region was the least preferred site of attachment probably due to high temperatures in the study area that can cause desiccation and death to ticks. In this study, goats and sheep were predominantly infected with *R. evertsi evertsi* attached to the anal region. This is consistent with a report from South Africa indicating that adult *R. evertsi evertsi* has a high preference for the perianal region or the groin of animal hosts [53]. The most predominant tick species infesting cattle, *A. variegatum*, had the udder/scrotum as a strong predilection site. This can be compared to studies in Cameroon and Burkina Faso where *A. variegatum* has a preference for the udder, reducing milk production and hindering animal growth [33, 35]. Furthermore, it conforms to a report that *A. variegatum* is found on the comparatively hairless regions of the host such as the ventral surface, genitalia, or underneath the tail [32]. Hunter species *H. rufipes* and *H. truncatum* had a preference for the anal region of cattle. Knowledge of the tick population within a region and their preferred attachment sites is essential in the formulation of efficient control strategies to curb the burden of infestation and disease transmission [52].

In tropical and subtropical regions, ticks are the most significant ectoparasites of livestock and cause significant economic losses both directly through bloodsucking and indirectly through their role as vectors of infectious pathogens [54]. This study also reaffirms that ticks continue to be a major concern and cause considerable obstacles to the production of livestock [55].

The current study did not analyze the distribution and predilection sites of ticks relative to the livestock breed or strain. This is recommended in future studies. It is also recommended that tick species such as *A. variegatum*, *H. rufipes*, and *H. truncatum* which show definite sites of predilection in attachment to cattle during one or more of their developmental stages, a local treatment targeting their preferred predilection sites could well offer a more suitable and economic means of control rather than dipping the entire livestock or treating the whole body with acaricides.

## 6. Conclusion

In this study, *A. variegatum* was the most predominant tick species infesting mostly cattle in the study area and had a preference for resting and feeding around the udder/scrotum. The study also indicated that the wet season was the period when adult tick infestation was high with the nymphal stages occurring in the dry season. Chemical control strategies should be designed based on knowledge of the bionomics, seasonal variation in species compositions, and the species-specific predilection sites on livestock.

## Figures and Tables

**Table 1:** Characteristics and distribution of sampled ticks.

	Total screened (%)	Nymph (*n* = 303)		Adult (*n* = 1247)	
		Ticks collected (%)	*p* value	Ticks collected (%)	*p* value
Host					
Cattle	243 (54.24)	303 (100)	—	1052 (84.36)	0.0931
Goat	85 (18.97)	—		13 (1.04)	
Sheep	120 (26.79)	—		182 (14.60)	
Sex					
Male	135 (30.13)	244 (80.53)	<0.001^*∗*^	444 (35.61)	0.3581
Female	313 (69.87)	59 (19.47)		803 (64.39)	
Age					
≤3 years	299 (66.74)	152 (50.17)	0.252	644 (51.64)	0.0595
>3 years	149 (33.26)	151 (49.83)		603 (48.36)	
Season					
Dry	276 (61.61)	303 (100)		410 (32.88)	<0.001^*∗*^
Wet	172 (38.39)	—	—	837 (67.12)	
Body part					
Udder/scrotum	448	177 (58.42)	<0.001^*∗*^	527 (42.26)	0.0529
Chest	448	77 (25.41)		258 (20.69)	
Anal	448	48 (15.84)		441 (35.37)	
Head	448	1 (0.33)		3 (0.24)	
Limbs	448	—		18 (1.44)	

^*∗*^Statistically significant.

**Table 2:** Risk factors for nymph tick infestation.

Characteristics	Estimate (95% CI)	Incidence rate ratio (95% CI)	*p* value
Animal sex			
Female vs. male	0.69 (0.26–1.11)	1.99 (1.30–3.05)	0.002^*∗*^
Age			
≤3 years vs. >3 years	0.04 (−0.30–0.38)	1.04 (0.74–1.46)	0.822
Body part			
Anal vs. chest	0.54 (0.07–1.00)	1.71 (1.07–2.72)	0.024^*∗*^
Anal vs. head	−0.27 (−2.47–1.92)	0.76 (0.08–6.83)	0.807
Anal vs. udder/scrotum	0.45 (0.003–0.90)	1.57 (1.00–2.46)	0.048^*∗*^

^*∗*^Statistically significant.

**Table 3:** Risk factors for adult tick infestation.

Characteristics	Estimate (95% CI)	Incidence rate ratio (95% CI)	*p* value
Animal sex			
Female vs. male	−0.09 (−0.28–0.11)	0.92 (0.75–1.11)	0.376
Season			
Wet vs. dry	0.65 (0.39–0.91)	1.92 (1.48–2.49)	<0.001^*∗*^
Age			
≤3 years vs. >3 years	−0.01 (−0.21–0.18)	0.99 (0.81–1.20)	0.901
Host			
Cattle vs. goat	0.18 (−0.59–0.95)	1.19 (0.55–2.58)	0.652
Cattle vs. sheep	0.22 (−0.09–0.53)	1.25 (0.91–1.71)	0.171
Body part			
Anal vs. chest	−0.03 (−0.35–0.29)	0.97 (0.71–1.33)	0.852
Anal vs. head	−1.32 (−2.65–0.004)	0.27 (0.07–1.00)	0.051
Anal vs. limbs	−0.38 (−1.04–0.27)	0.68 (0.35–1.31)	0.253
Anal vs. udder/scrotum	−0.01 (−0.30–0.27)	0.99 (0.74–1.31)	0.921

^*∗*^Statistically significant.

**Table 4:** Seasonal distribution of tick species.

Tick species	Total no. (%)	Nymph			Adult		
		Wet	Dry	*p*-value	Wet	Dry	*p* value
*Amblyomma variegatum*	976 (62.98)	—	201	—	741	34	<0.001^*∗*^
*Hyalomma rufipes*	169 (10.9)	—	—	—	16	153	0.6497
*Hyalomma truncatum*	121 (7.81)	—	5	—	6	110	0.19
*Rhipicephalus evertsi evertsi*	181 (12)	—	—	—	74	112	0.4343
*Rhipicephalus* (*Boophilus*) sp.	86 (5.35)	—	83	—	—	—	—
*Rhipicephalus geigyi*	14 (0.9)	—	14	—	—	—	—
*Rhipicephalus sanguineus* (s.l.)	3 (0.06)	—		—	—	1	—

**Table 5:** Association of tick species with the preferred site of attachment on livestock.

Tick species	Adult (*n* = 1247)					Nymph (*n* = 303)			
	Part of attachment	Total number of animals	Ticks collected *N* (%)	Mean ± SE	*p* value	Total number of animals	Ticks collected *N* (%)	Mean ± SE	*p* value
*Amblyomma variegatum*	Anal	20	34 (2.73)	1.7 ± 0.40	<0.001^*∗*^	12	29	2.42 ± 0.79	0.2381
	Chest	66	253 (20.29)	3.83 ± 0.37		12	51	4.25 ± 0.87	
	Head	3	3 (0.24)	1		0	—	—	
	Limbs	6	13 (1.04)	2.17 ± 0.60		0	—	—	
	Udder/scrotum	109	472 (37.85)	4.33 ± 0.30		30	121	4.03 ± 0.56	

*Hyalomma rufipes*	Anal	69	142 (11.39)	2.06 ± 0.21	0.3291	0	—	—	—
	Chest	1	2	2		0	—	—	
	Head	0	0	0		0	—	—	
	Limbs	1	1	1		0	—	—	
	Udder/scrotum	14	24	1.71 ± 0.37		0	—	—	

*Hyalomma truncatum*	Anal	33	81	2.45 ± 0.30	<0.001^*∗*^	—	—	—	—
	Chest	3	3	1		1	5	5	
	Head	0	—	—		—	0	—	
	Limbs	1	4	4		—	0	—	
	Udder/scrotum	20	28	1.4 ± 0.11		—	0	—	

*Rhipicephalus evertsi evertsi*	Anal	51	184	3.61 ± 0.44	0.6002	0	—	—	—
	Chest	0	—	—		0	—	—	
	Head	0	—	—		0	—	—	
	Limbs	0	—	—		0	—	—	
	Udder/scrotum	1	2	2		0	—	—	

*Rhipicephalus* (*Boophilus*) sp.	Anal	0	—	—	—	11	14	1.27 ± 0.14	0.2586
	Chest	0	—	—		5	20	4 ± 1.76	
	Head	0	—	—		1	1	1	
	Limbs	0	—	—		0	—	—	
	Udder/scrotum	0	—	—		14	48	3.42 ± 0.88	

*Rhipicephalus geigyi*	Anal	0	—	—	—	3	5	5	0.0856
	Chest	0	—	—		1	1	1	
	Head	0	—	—		0	—	—	
	Limbs	0	—	—		0	—	—	
	Udder/scrotum	0	—	—		2	8	4 ± 2	

*Rhipicephalus sanguineus* (s.l.)	Anal	0	—	—	—	0	—	—	—
	Chest	0	—	—		0	—	—	
	Head	0	—	—		0	—	—	
	Limbs	0	—	—		0	—	—	
	Udder/scrotum	1	1	1		0	—	—	

## Data Availability

All the data supporting this study are included in the article.
